# Supplementary material for: Water-soluble vidarabine derivatives alleviate catecholamine-induced heart failure and arrhythmia without impairing cardiac function in mice
Source: PLoS One. 2025 Aug 20;20(8):e0330507. doi: 10.1371/journal.pone.0330507 (PMC12367191; doi:10.1371/journal.pone.0330507)
Supplement: S1 Data — (PDF) [file pone.0330507.s001.pdf]

S1 Data

Water-soluble vidarabine derivatives alleviate catecholamine-induced heart failure and arrhythmia without impairing cardiac function in mice

Kenji Suita [1], Yoshio Hayakawa [1,2], Yujiro Hoshino [3], Wenqian Cai [4], Reiko Kurotani [5], Yoshiki Ohnuki [1], Yasumasa Mototani [1], Yoshihiro Ishikawa [6], Satoshi Okumura [1*]

[1] Department of Physiology, Tsurumi University School of Dental Medicine, Yokohama 230-8501, Japan.
[2] Department of Dental Anesthesiology, Tsurumi University School of Dental Medicine, Yokohama 230-8501, Japan.
[3] Graduate School of Environment and Information Sciences, Yokohama National University, Tokiwadai, Hodogaya-ku, Yokohama 240-8501, Japan.
[4] Heart Center and Guangzhou Institute of Pediatrics, Guangzhou Women and Children's Medical Center, Guangzhou Medical University, 9 JinSui Rd, Guangzhou, Guangdong 510120, China.
[5] Graduate School of Science and Engineering, Faculty of Engineering, Yamagata University, Yonezawa, Yamagata 992-8510, Japan
[6] Cardiovascular Research Institute, Yokohama City University Graduate School of Medicine, 3-9 Fukuura, Kanazawa-ku, Yokohama, 236-0004, Japan.

* Corresponding author.
Department of Physiology, Tsurumi University School of Dental Medicine, 2-1-3 Tsurumi, Tsurumi-ku, Yokohama 230-8501, Japan.
E-mail address: okumura-s@tsurumi-cu.ac.jp

S1 Fig

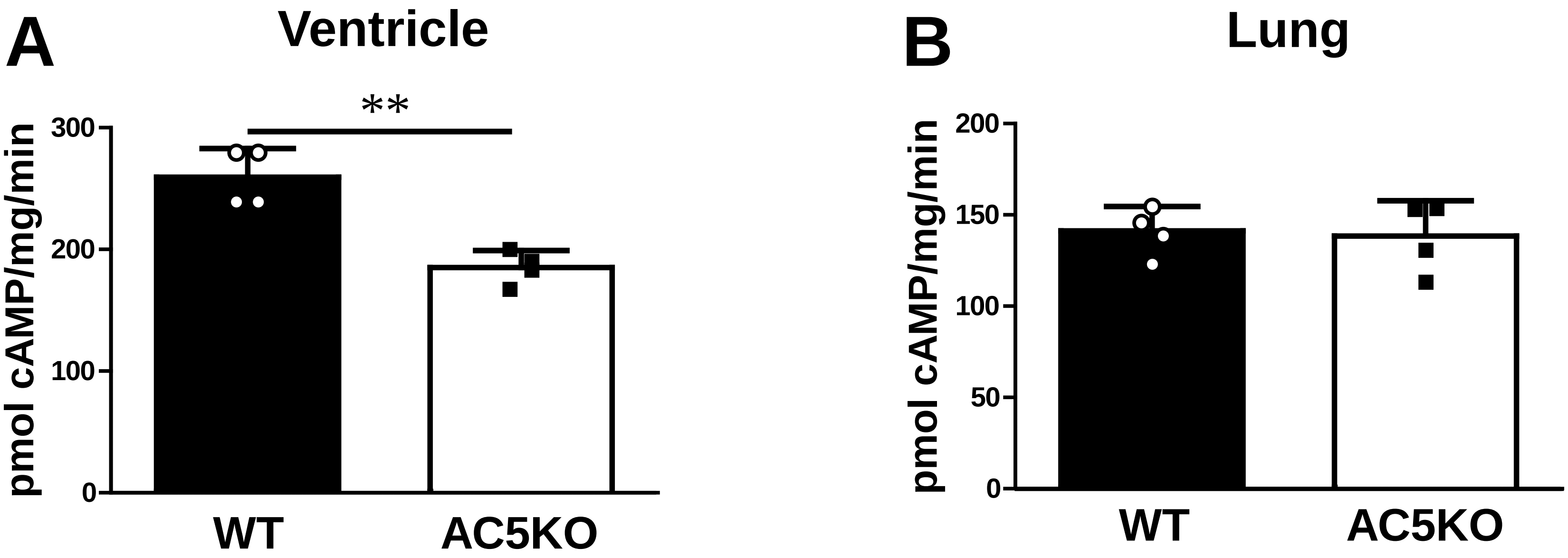

S1 Fig. AC activity in ventricle and lung of AC5KO mice.
(A) The AC activity in the ventricle was significantly decreased in AC5KO, compared to WT mice. **(B)** The AC activity of lung was similar in WT and AC5KO. Statistical significance was analyzed by Student's unpaired *t*-test. $^{**}P < 0.01$. Data are presented as mean ± SD and dots show individual data from four WT and four AC5KO mice ($n = 4$).

S2 Fig

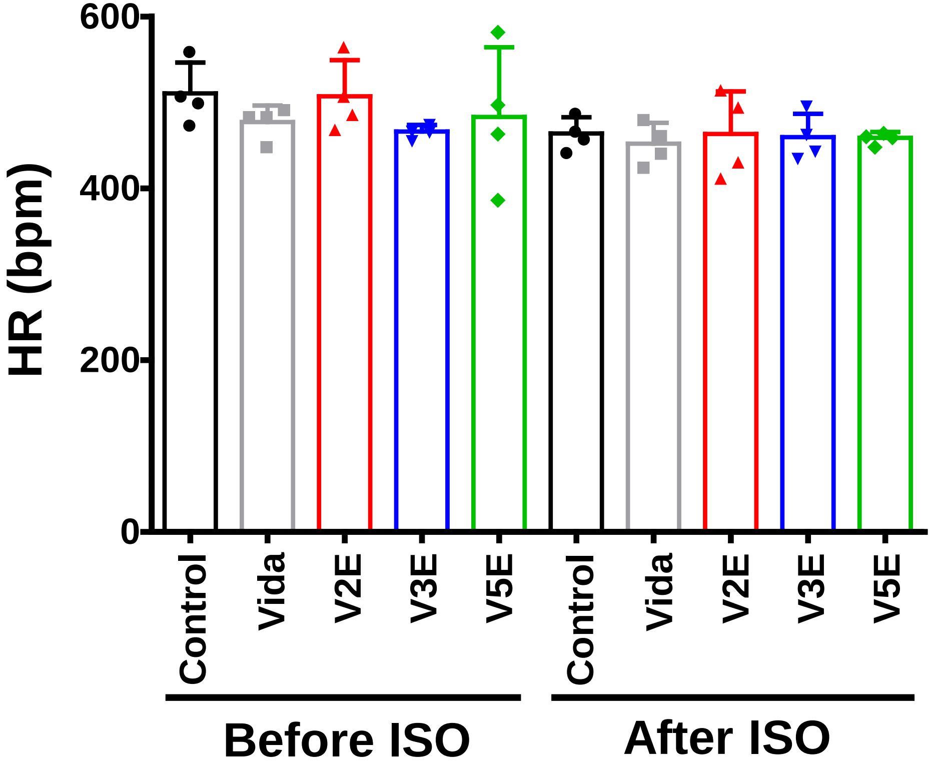

S2 Fig. Effects of Vida, V2E, V3E and V5E on heart rate after chronic ISO infusion. Echocardiography was performed to examine the effects of Vida, V2E, V3E and V5E on heart rate (HR) before (*left*) and after (*right*) chronic ISO infusion. HR was similar among the five groups before (*left*) and after (*right*) chronic ISO infusion. Statistical significance was analyzed by two-way ANOVA with Bonferroni's *post hoc* test. Data are presented as mean ± SD and dots show individual data from four WT mice ($n = 4$).

S3 Fig

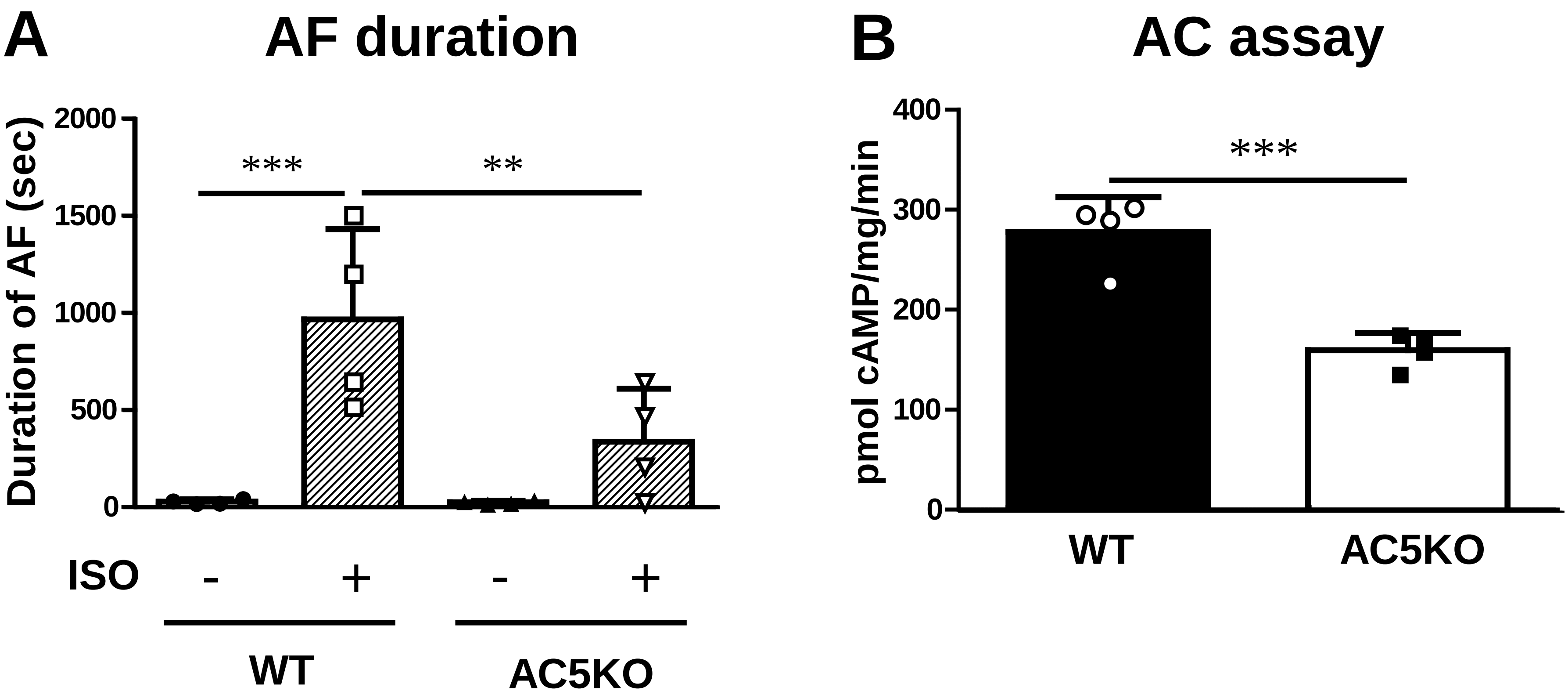

S3 Fig. Effects of AC5 gene deficiency on ISO-elongated AF and atrial AC activity.
(A) The duration of ISO-elongated AF induced by transesophageal atrial burst pacing was significantly decreased in AC5KO compared with WT. (**B**) The AC activity was significantly decreased in AC5KO atrium compared with WT atrium. Statistical significance was analyzed by two-way ANOVA with Bonferroni's *post hoc* test in **(A)** and Student's unpaired *t*-test in **(B)**. $^{**}P < 0.01$, $^{***}P < 0.001$. Data are presented as mean ± SD and dots show individual data from four WT and four AC5KO mice ($n = 4$).

S4 Fig

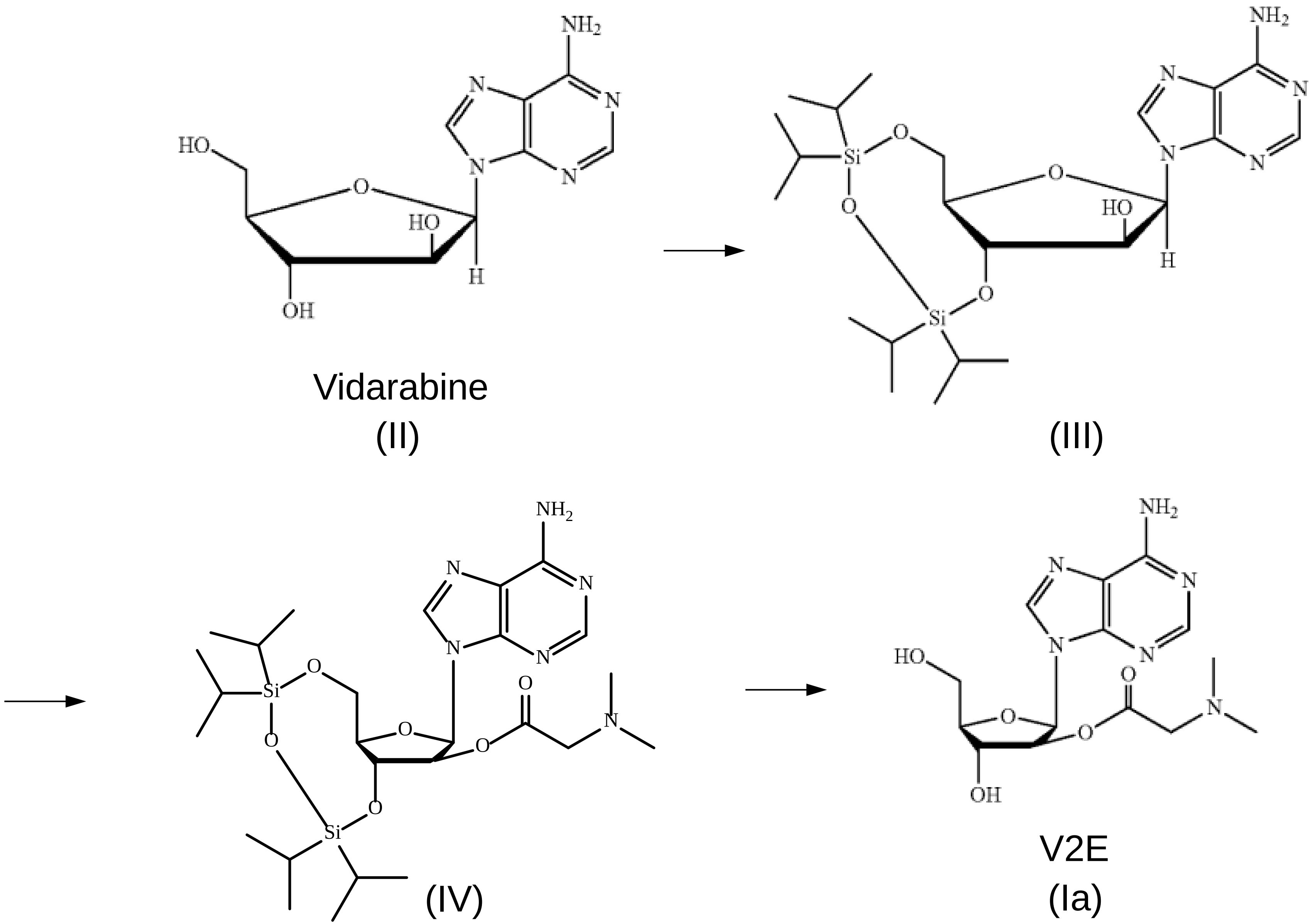

S4 Fig. Synthesis of V2E.

S5 Fig

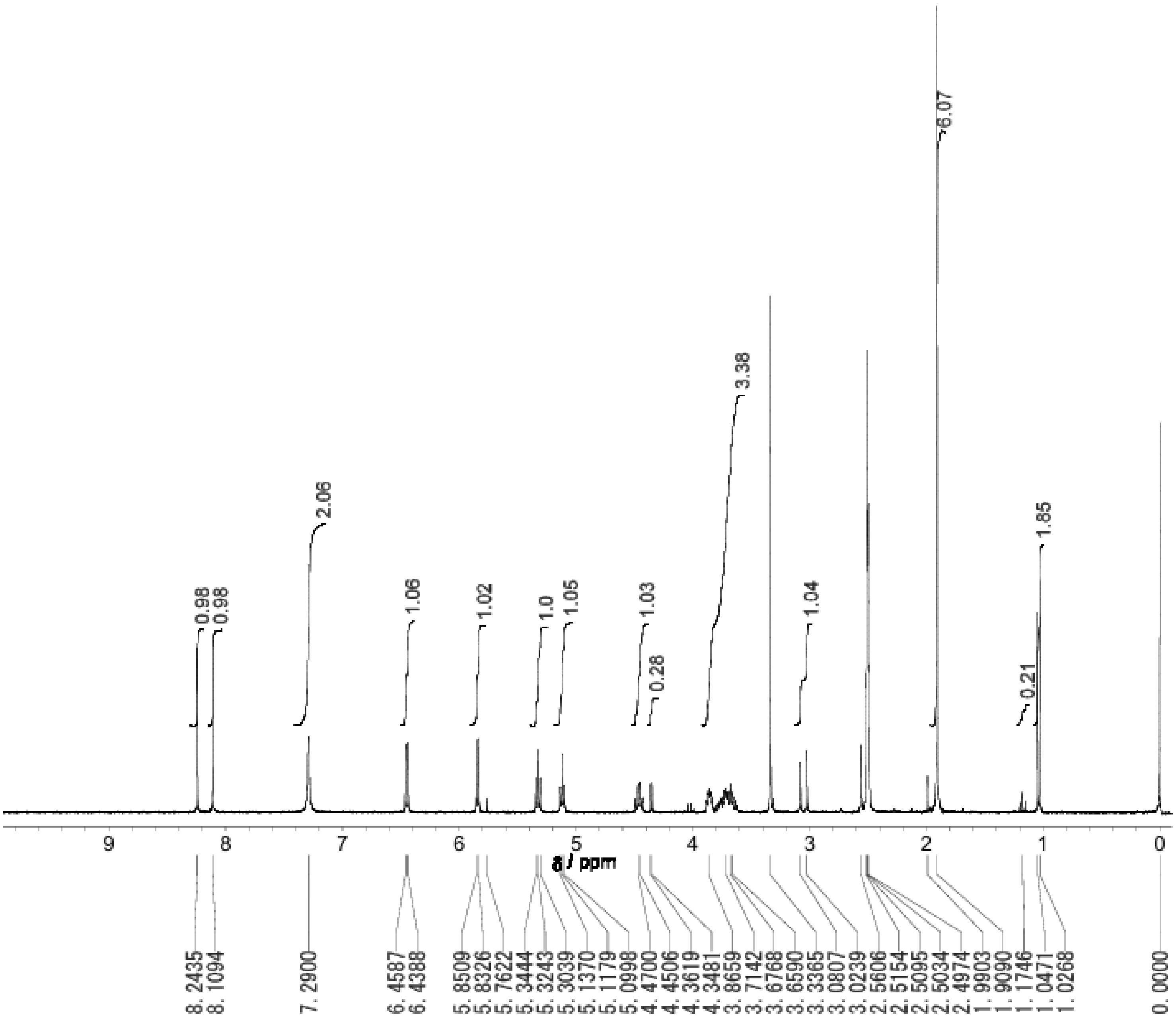

S5 Fig. ^{1}H NMR spectrum of V2E.

S6 Fig

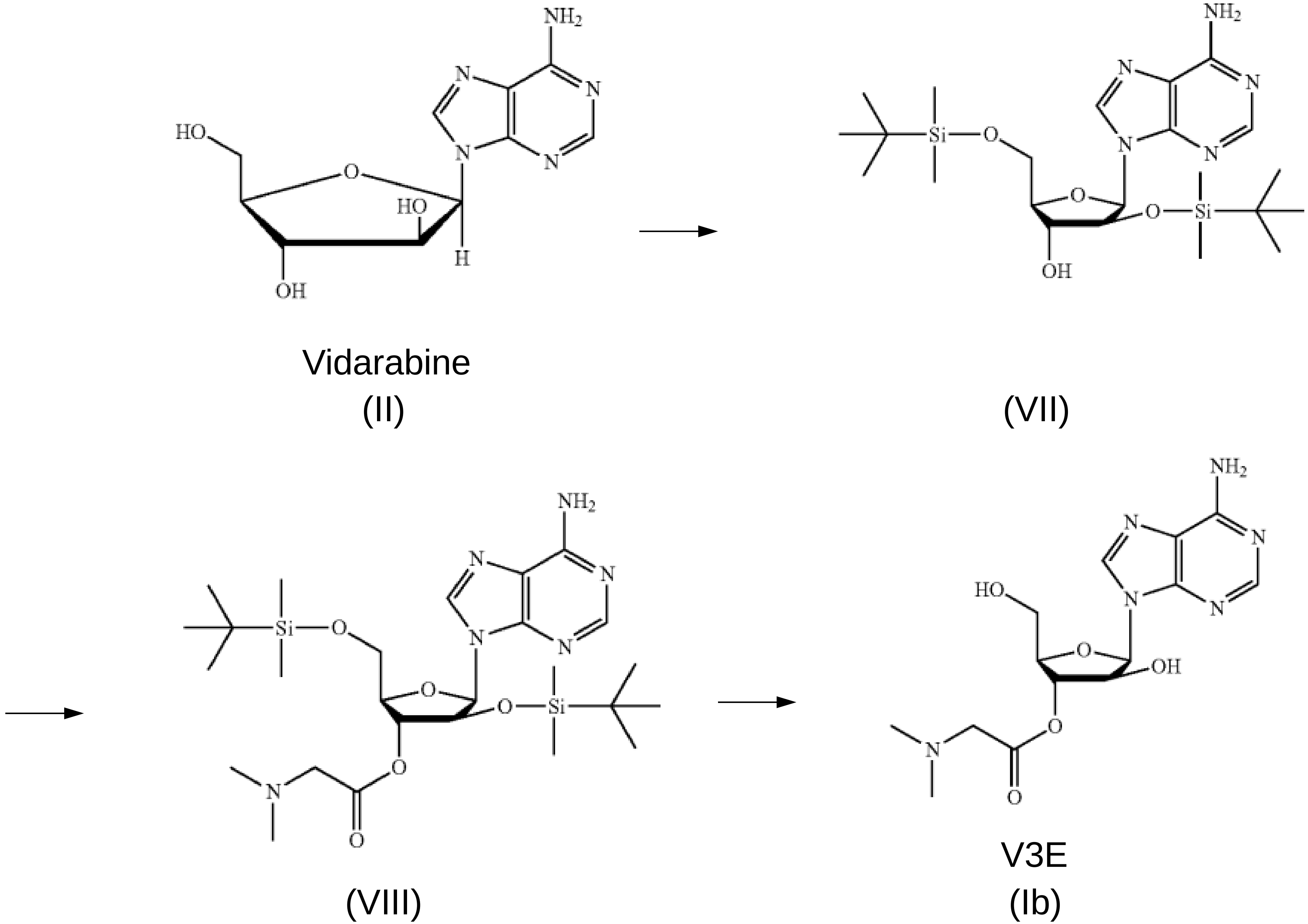

S6 Fig. Synthesis of V3E.

S7 Fig

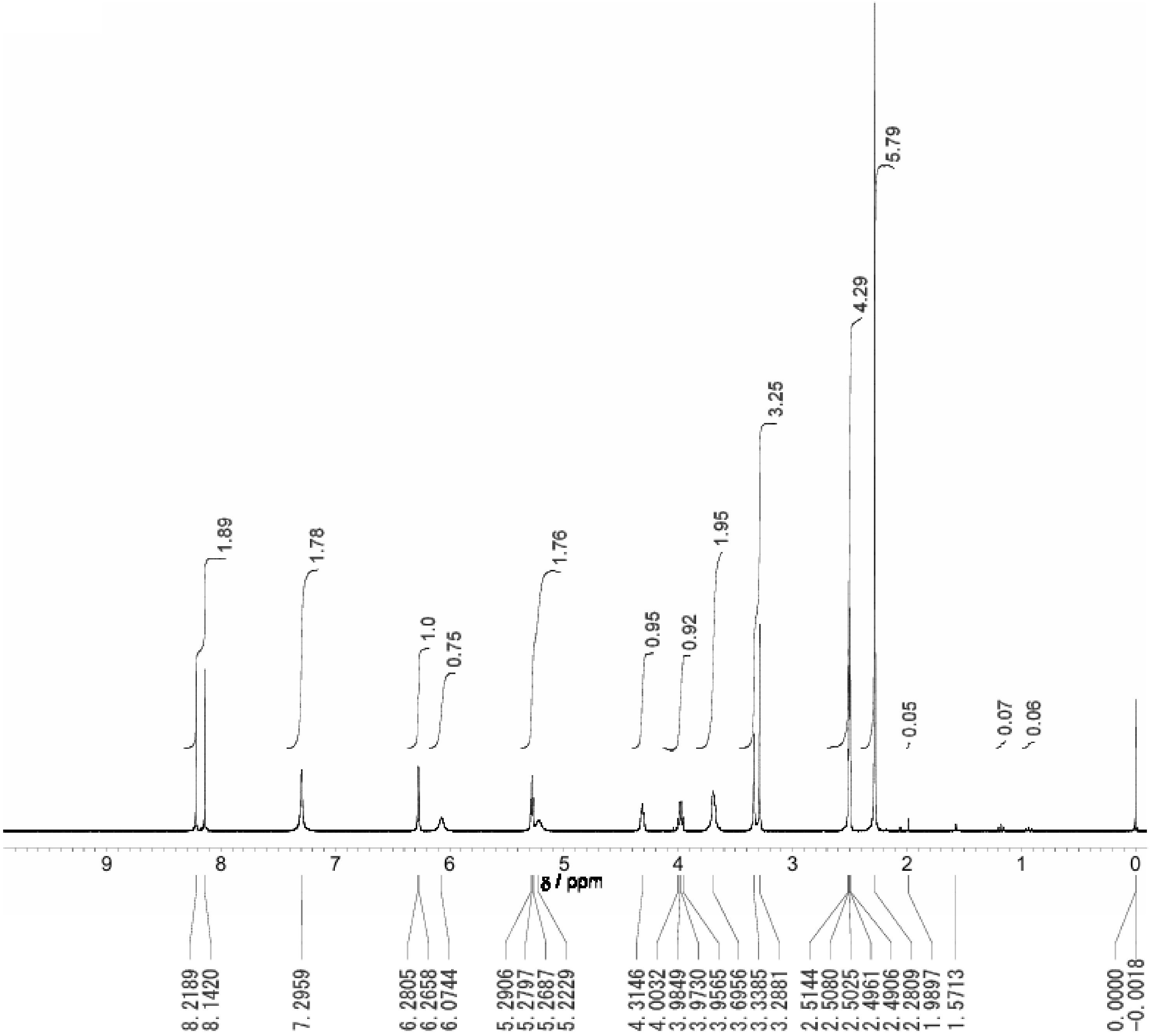

S7 Fig. ^{1}H NMR spectrum of V3E.

S8 Fig

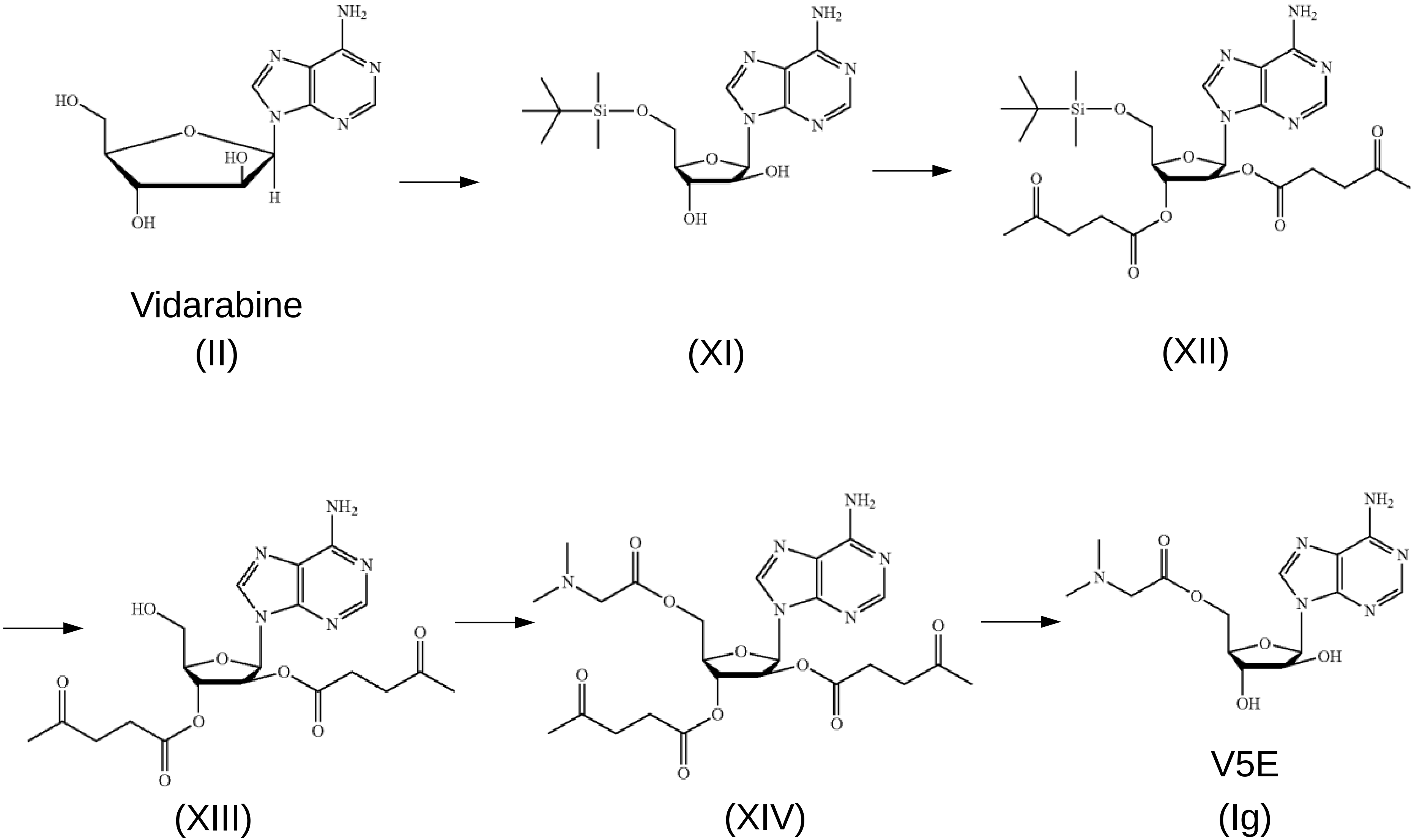

S8 Fig. Synthesis of V5E.

S9 Fig

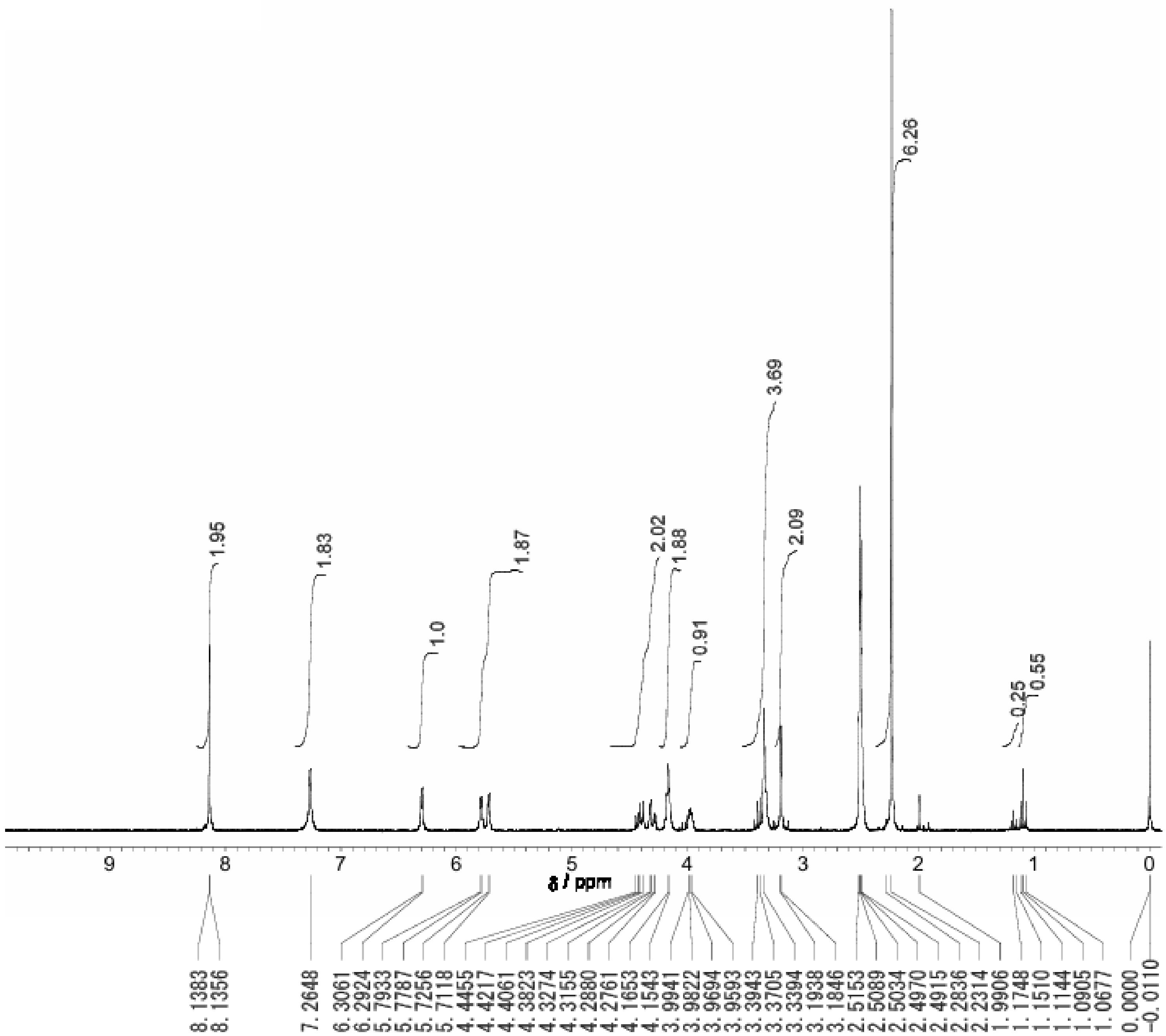

S9 Fig. ^{1}H NMR spectrum of V5E.

S10 Fig

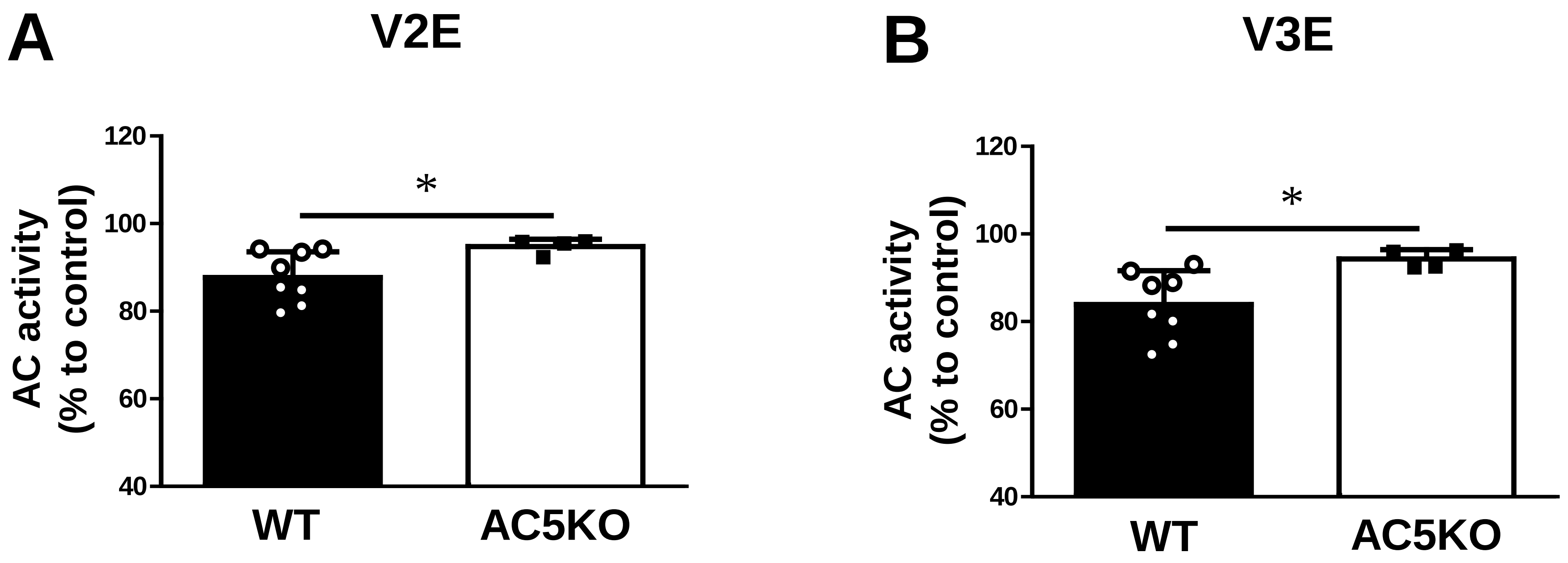

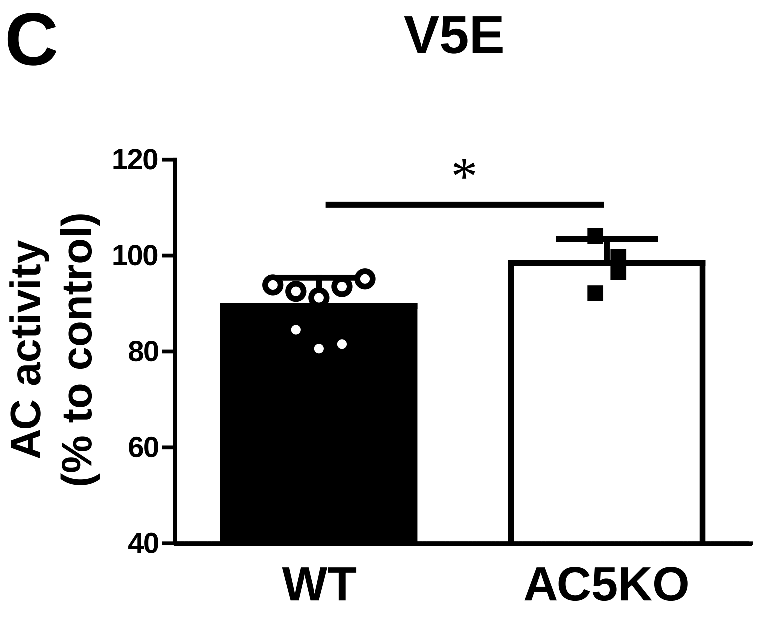

S10 Fig. Comparison of the inhibitory effects of V2E, V3E and V5E on AC activity in the heart of WT and AC5KO mice.

The inhibitory effects of V2E **(A)**, V3E **(B)** and V5E **(C)** at 10 μmol/L on the ISO (50 μmol/L)-stimulated AC activity of AC5KO mice were slightly but significantly smaller than those in WT mice. The AC activity without inhibitor was set to 100 in each case. Statistical significance was analyzed by Student's unpaired *t*-test. $^{*}P < 0.05$. Data are presented as mean ± SD and dots show individual data from eight WT mice and four AC5KO mice (WT: n = 8, AC5KO: n = 4).

S11 Fig

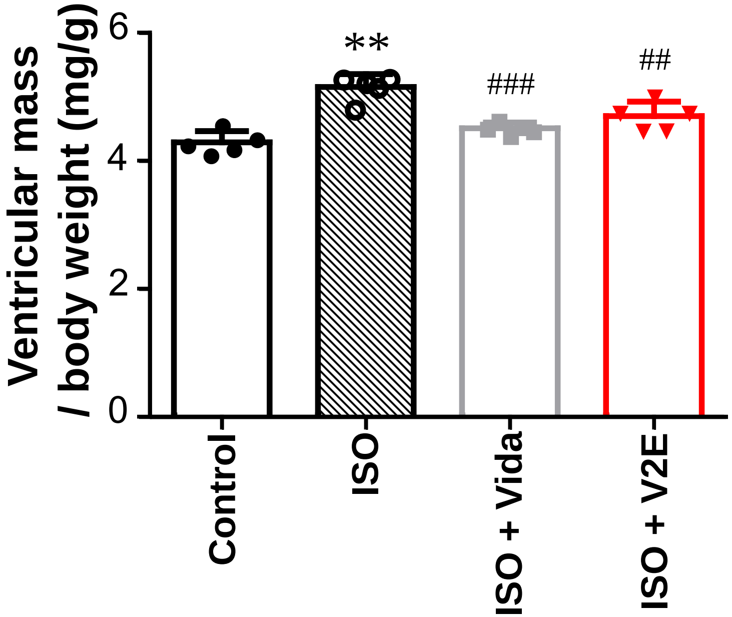

S11 Fig. Effects of Vida and V2E on the ventricular hypertrophy induced by chronic ISO infusion. ISO (60 mg/kg/day) with Vida (15 mg/kg/day) or V2E (19.7 mg/kg/day) was subcutaneously administered to WT mice via an osmotic mini-pump for 7 days. Ventricular hypertrophy was evaluated in terms of ventricular mass to body weight ratio. Vida and V2E significantly suppressed the ISO-induced ventricular hypertrophy. Statistical significance was analyzed by one-way ANOVA with Tukey-Kramer's *post hoc* test. $^{**}P < 0.01$ vs control; $^{\#\#}P < 0.01$, $^{\#\#\#}P < 0.001$ vs ISO. Data are presented as mean ± SD and dots show individual data from five WT mice ($n = 5$).

S12 Fig

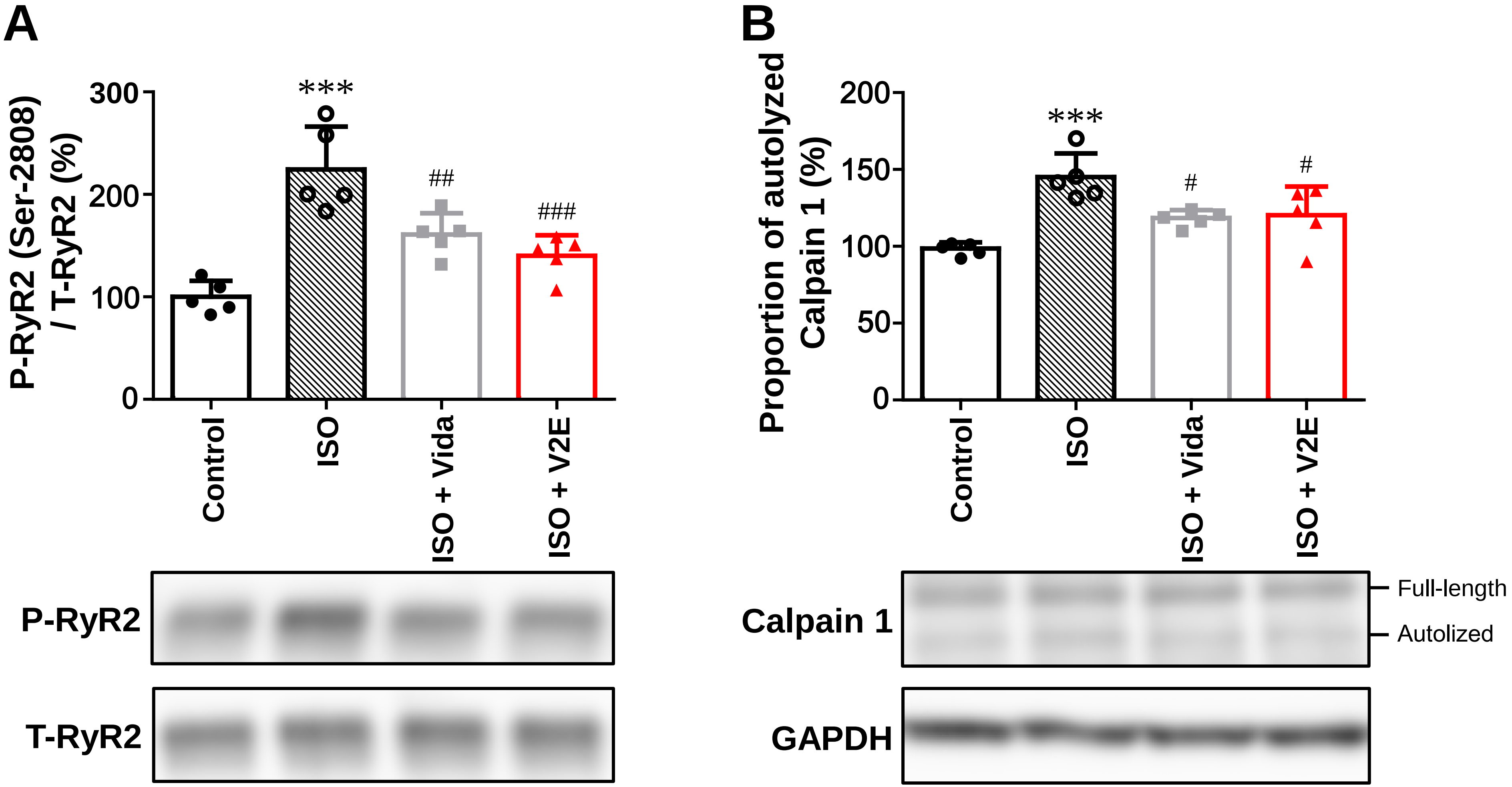

S12 Fig. Effects of Vida and V2E on cellular Ca^{2+} homeostasis in ventricle.

ISO (60 mg/kg/day) with Vida (15 mg/kg/day) or V2E (19.7 mg/kg/day) was subcutaneously administered to WT mice via an osmotic mini-pump for 7 days. **(A)** The RyR2 phosphorylation at Ser-2808 was significantly increased in the ventricle of the ISO group as compared to the control group, but the increase was attenuated in the ISO + Vida and ISO + V2E groups. **(B)** The calculated calpain 1 fractional activation index (*i.e.*, percentage of autolyzed calpain 1) was significantly greater in the ISO group than in the control group, but this was not the case in the ISO + Vida and ISO + V2E groups. Statistical significance was analyzed by one-way ANOVA with Tukey-Kramer's *post hoc* test. $^{***}P < 0.001$ vs control; $^{\#}P < 0.05$, $^{\#\#}P < 0.01$, $^{\#\#\#}P < 0.001$ vs ISO. Data are presented as mean ± SD and dots show individual data from five WT mice (*n* = 5).
