# Supplementary material for: Water-soluble vidarabine derivatives alleviate catecholamine-induced heart failure and arrhythmia without impairing cardiac function in mice
Source: PLoS One. 2025 Aug 20;20(8):e0330507. doi: 10.1371/journal.pone.0330507 (PMC12367191; doi:10.1371/journal.pone.0330507)
Supplement: S2 Data — (PDF) [file pone.0330507.s002.pdf]

S2 Data

Water-soluble vidarabine derivatives alleviate catecholamine-induced heart failure and arrhythmia without impairing cardiac function in mice

Kenji Suita [1], Yoshio Hayakawa [1,2], Yujiro Hoshino [3], Wenqian Cai [4], Reiko Kurotani [5], Yoshiki Ohnuki [1], Yasumasa Mototani [1], Yoshihiro Ishikawa [6], Satoshi Okumura [1*]

[1] Department of Physiology, Tsurumi University School of Dental Medicine, Yokohama 230-8501, Japan.
[2] Department of Dental Anesthesiology, Tsurumi University School of Dental Medicine, Yokohama 230-8501, Japan.
[3] Graduate School of Environment and Information Sciences, Yokohama National University, Tokiwadai, Hodogaya-ku, Yokohama 240-8501, Japan.
[4] Heart Center and Guangzhou Institute of Pediatrics, Guangzhou Women and Children's Medical Center, Guangzhou Medical University, 9 JinSui Rd, Guangzhou, Guangdong 510120, China.
[5] Graduate School of Science and Engineering, Faculty of Engineering, Yamagata University, Yonezawa, Yamagata 992-8510, Japan
[6] Cardiovascular Research Institute, Yokohama City University Graduate School of Medicine, 3-9 Fukuura, Kanazawa-ku, Yokohama, 236-0004, Japan.

* Corresponding author.
Department of Physiology, Tsurumi University School of Dental Medicine, 2-1-3 Tsurumi, Tsurumi-ku, Yokohama 230-8501, Japan.
E-mail address: okumura-s@tsurumi-cu.ac.jp

Original blots of S12A

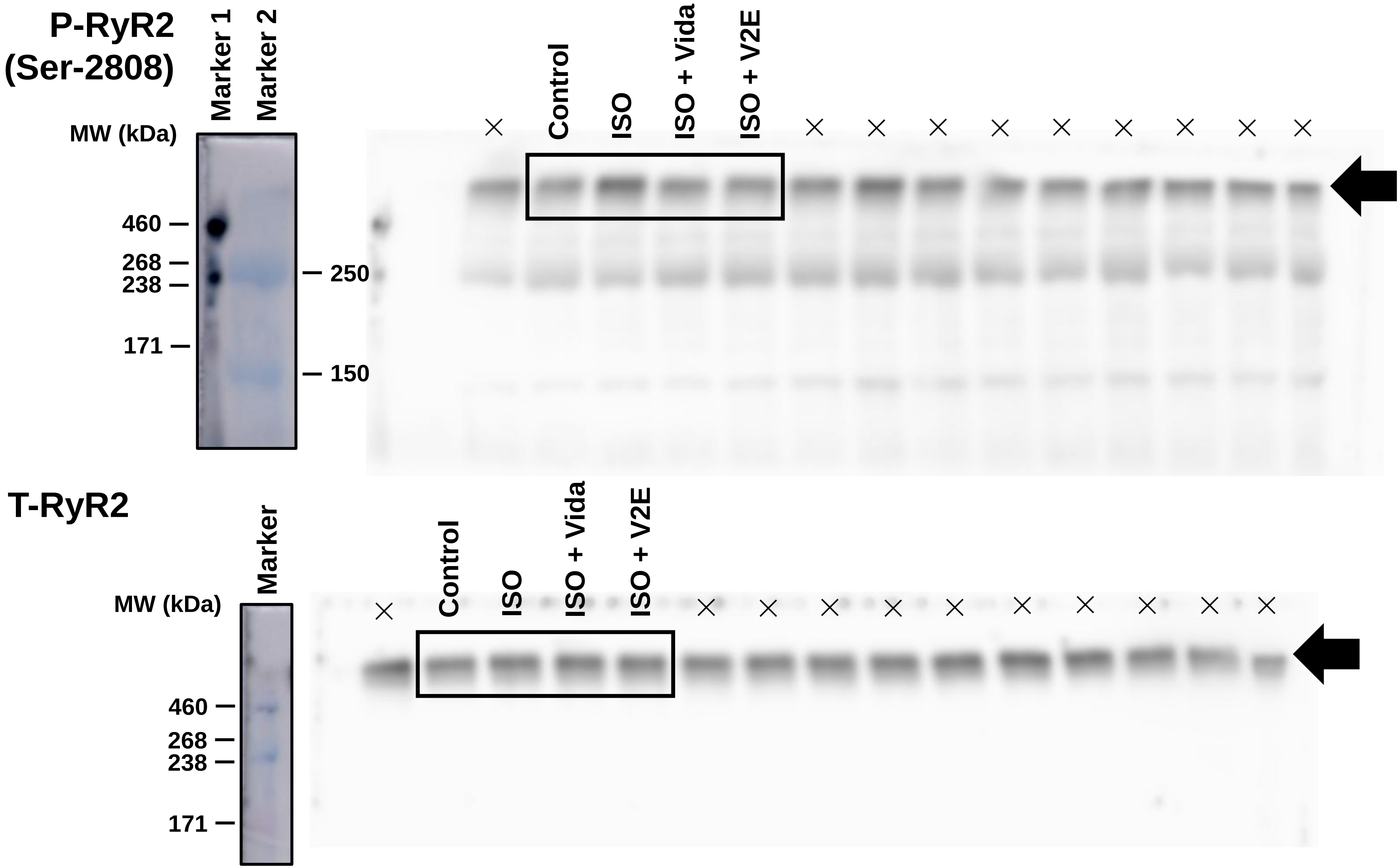

Representative full-length immunoblots of **S12A Fig**. The amounts of phosphorylated RyR2 at serine-2808 and total RyR2 are shown. The black-line box indicated by arrow in each blot corresponds to the cropped parts that are shown in the main article. MW: molecular weight.

Original blots of S12B

Calpain 1

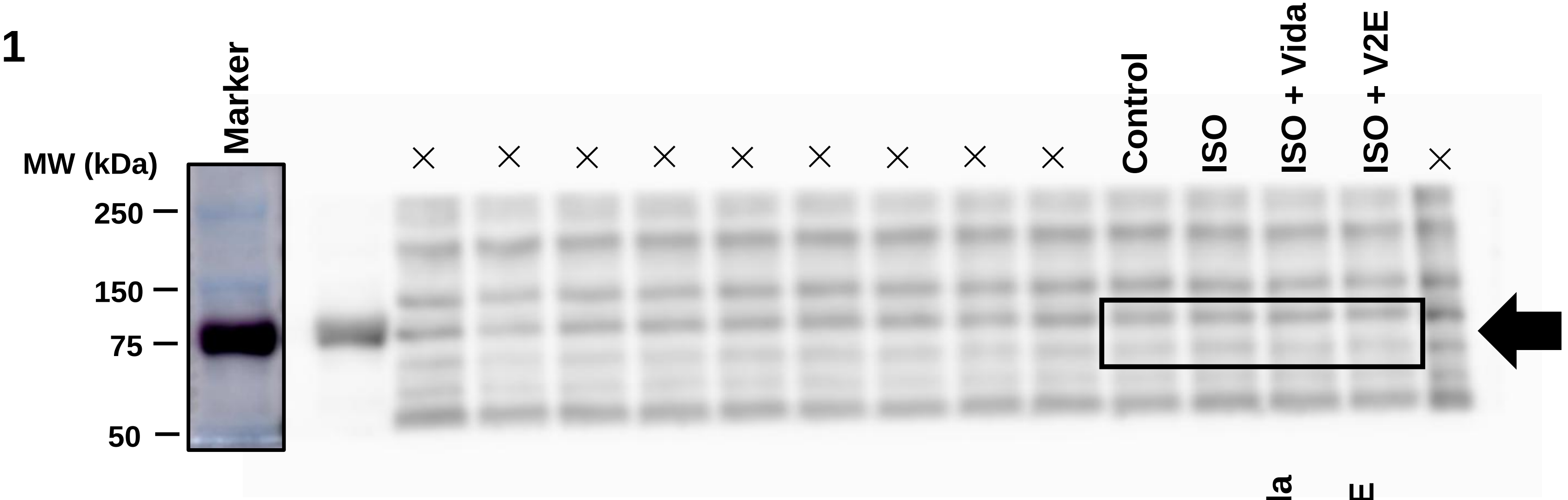

GAPDH

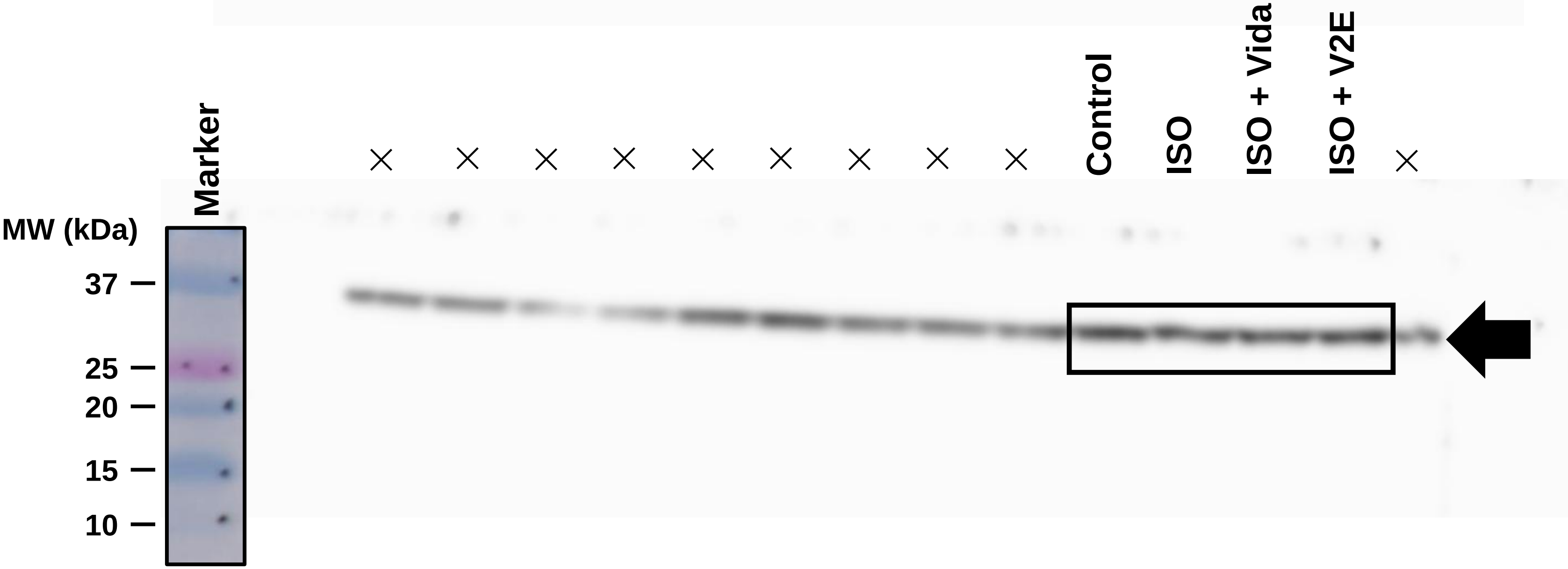

Representative full-length immunoblots of **S12B Fig**. The amount of full-length calpain 1 (78kDa, upper band), autolyzed calpain 1 (74 kDa, lower band) and GAPDH are shown. The black-line box indicated by arrow in each blot corresponds to the cropped parts that are shown in the main article. MW: molecular weight.
