# Supplementary material for: Water-soluble vidarabine derivatives alleviate catecholamine-induced heart failure and arrhythmia without impairing cardiac function in mice
Source: PLoS One. 2025 Aug 20;20(8):e0330507. doi: 10.1371/journal.pone.0330507 (PMC12367191; doi:10.1371/journal.pone.0330507)
Supplement: Attachment — Submitted filename: PLOS One.pdf [file pone.0330507.s004.pdf]

Here are some General comments on the manuscript:

- The introduction effectively sets the stage for the study by highlighting the importance of heart failure and the role of the autonomic nervous system. However, some sentences are long and complex, which may make it harder for readers to follow. Consider breaking them into shorter, more digestible sentences.
- While HF is mentioned as a leading cause of mortality, a brief explanation of its prevalence, types, and associated symptoms would provide readers with essential background. This will help underscore the urgency of developing new treatments.
- The statement about the autonomic nervous system could benefit from clarification. Specify how its long-term activation adversely affects cardiac function. Including a brief description of the sympathetic nervous system's role would help.
- When discussing β-blockers, it would be helpful to explain how they work and why their adverse effects are particularly problematic in older patients. For example, they can mention how β-blockers manage heart rate and contractility but can lead to complications in patients with coexisting pulmonary conditions.
- When introducing AC5, its specificity to the heart was mentioned but an elaboration on its role in cardiac function and how it differs from other isoforms is important. This will enhance understanding of why selective inhibition of AC5 is a viable therapeutic strategy.
- The discussion about vidarabine’s solubility issues is critical. Consider including specific examples of how poor solubility impacts clinical administration and patient outcomes, emphasizing the need for improved formulations.
- When stating that direct inhibition of AC could prevent pathological β-AR activation, briefly mention the potential physiological benefits of this approach. This could include improved cardiac function or reduced arrhythmia risk.
- Previous studies on AC5KO mice were mentioned. Summarizing key findings from these studies (e.g., effects on longevity and stress resistance) will strengthen the rationale for targeting AC5 in the current work.
- It was noticed that the transition to discussing vidarabine derivatives is abrupt. Consider a bridging sentence that connects the limitations of vidarabine to the need for developing these new derivatives. Explain how modifying the structure could overcome previous obstacles.
- In the objectives of the study, the final paragraph outlines the focus on vidarabine derivatives but could more explicitly state the main objectives of the study. Clearly define what you aim to achieve with this research, such as improving solubility and reducing side effects.

Here are some Specific comments on the manuscript:

- The statistic stating that HF affects "an estimated 26 million people and contributes to more than 1 million hospitalizations" could be enhanced by citing specific sources. Ensure that the references are up-to-date and relevant.
- The mention of β-blockers and their limitations is important, but it might be beneficial to briefly explain how they currently work in HF management before discussing their adverse effects.
- The discussion on the synthesis of vidarabine derivatives (V2E, V3E, V5E) could be more detailed. Providing some background on how these specific modifications improve solubility would be helpful.
- The findings on the efficacy of V2E, V3E, and V5E are significant, however, adding a comparison to existing therapies could help emphasize their potential advantages.
- The section on AC isoforms (AC5 and AC6) is informative. Consider summarizing the key implications of the findings related to these isoforms for a broader audience who may not be familiar with this area.
- When discussing how V2E, V3E, and V5E selectively inhibit AC5, it would be helpful to provide some mechanistic insights. A diagram could be useful to illustrate these interactions and effects.
- Referencing figures consistently throughout the results aids in visual comprehension and supports the textual claims. Ensure that the figures are clearly labeled and match the referenced data.
- The findings on fibrosis and apoptosis are well-articulated. Including a brief discussion on the underlying mechanisms by which V2E, V3E, and V5E exert their protective effects could add depth to your results.
- If possible, use tables to summarize key data points, such as solubility levels, AC activity measurements, and cardiac function parameters to improve readability and quick reference for readers.
- Explain how poor solubility impacts clinical use, such as the need for intravenous administration and associated complications like fluid overload. This highlights the urgency for improved formulations.
- Explain why substituting with (dimethylamino)acetic acid at specific positions enhances solubility. A brief description of the chemical properties that affect solubility could clarify this for readers not familiar with medicinal chemistry.
- End with a paragraph discussing potential future studies. This could include clinical trials, investigations into long-term effects, or comparisons with existing therapies. Highlight any specific patient populations that may benefit most from these new compounds.
