# Supplementary material for: Water-soluble vidarabine derivatives alleviate catecholamine-induced heart failure and arrhythmia without impairing cardiac function in mice
Source: PLoS One. 2025 Aug 20;20(8):e0330507. doi: 10.1371/journal.pone.0330507 (PMC12367191; doi:10.1371/journal.pone.0330507)
Supplement: Attachment — Submitted filename: PLOS ONE Suita-08-add RS correction-05.pdf [file pone.0330507.s006.pdf]

Reviewer #1

The manuscript appears to be well-written in terms of technical language and clarity. However, there are a few areas where slight improvements can enhance readability:

Criticism-1:
Sentence Structure: Some sentences are quite long and could be split for clarity. Shorter sentences can make the text easier to follow.

Response-1:
Thank you for the suggestion. We have re-checked the manuscript and have split sentences that seemed particularly long or complex (see gray-shaded portions of the revised manuscript with tracked changes).

Criticism-2:
Consistency: Ensure that terms and abbreviations are used consistently throughout the manuscript. For example, if you introduce a term like “AC5” or “ADA”, make sure it is consistently abbreviated after the first mention.

Response-2:
Done as requested.

Criticism-3:
Punctuation: Review for proper punctuation, especially in complex sentences. Comma can help clarity meaning and improve flow.

Response-3:
We have checked the punctuation throughout the manuscript. Please also see our response to Criticism-1.

Criticism-4:
Technical Jargon: while the audience may be familiar with specific terms, consider briefly defining less common jargon or acronyms when first introduced.

Response-4:
1) Vaughan Williams classifications

We incorporated the following explanation (underlined) in the revised manuscript (Page 6, Lines 9-11).
---in the Vaughan Williams classification, which categorizes antiarrhythmic drugs into class IA, class IB, class IC, class II, class III, class IV and class V according to their mechanism of action, ---

2) Osmotic mini-pump
We incorporated the following sentences in the method section of the revised manuscript (Page 14, Lines 16-18)

Alzet osmotic mini-pumps are small, implantable pumps used for research in mice, rats, and other laboratory animals, enabling accurate and continuous dosing of unrestrained laboratory animals.

3) P-site inhibitor
We incorporated the following explanation (underlined) in the revised manuscript (Page 22, Lines 6-7).
---P-site inhibitors, which are adenosine analogs that inhibit AC catalytic activity in a non- or uncompetitive manner with respect to ATP [1], possess a pharmacophore structure---

Criticism-5:
Transition: Use transitional phrases to improve the flow between sections and ideas, helping guide the reader through the results and discussions.

Response-5:
We have included linking text to improve the flow where it seemed desirable (see gray-shaded portions of the revised manuscript with tracked changes).

Reviewer #2:

Authors tackled to prevent from heart failure and arrhythmia by specifically targeting adenylyl cyclase isoform, while avoiding cardiac function suppression. First, they synthesized derivatives (V2E, V3E and V5E) of vidaravine, by the substitution 2’, 3’ and 5’position of arabinose with a (dimethylamino) acetic acid group, to improve its

poor solubility. They confirmed an increased solubility of V2E, V3E and V5E compared to vidarabine. Further experimentally showed V2E, V3E and V5E reduced the 1) development of heart failure and 2) susceptibility to atrial fibrillation using mouse model.
Overall idea is interesting, and approach is proper. However, I believe current manuscript needs visual improvement (type of graphs) and clarification, including following points/questions/comments.

Introduction.

<u>Criticism-1:</u>

Authors claimed that “AC5 is expressed specifically in the heart, with little expression in the lung [5]”. However, according to cited literature, AC5 is detected in the heart at compatible level to brain. Therefore, authors either modify the above-mentioned claim or include additional reference to support AC5 expression is specific to heart. Otherwise, have a discussion on this point.

<u>Response-1:</u>

We incorporated the following phase with new references (Page 7, Lines 6-7).

---and among them, type 5 AC (AC5) is the major AC isoform in adult cardiac tissue. It is expressed at extremely low levels in other organs, except for the brain [2, 3].

Method section.

<u>Criticism-2:</u>

Even through it is understandable from the study, authors should clearly describe animal species that was used in the study, otherwise it is unclear from their “animals” subsection.

<u>Response-2:</u>

We incorporated the following sentences in the method section of the revised manuscript (Page 10, Lines 7-10).

The previously reported AC5KO mice were repeatedly backcrossed more than 7 times on a C57BL/6 background [4]. Neonatal Wister rats were used for the preparation of primary cell culture in this study (Sankyo Labo Service Corporation, Inc., Tokyo, Japan).

Criticism-3:

Also, please include number of animals used in this study per group per experiments, as well as their sex.

Response-3:

We incorporated the required information (number of animals in each study) in the Figure legends of the revised manuscript (Pages 56-61). We also noted that we used male mice in the method section of the revised manuscript (Page 10, Line 5).

Criticism-4:

Authors missing subsection for “cell culture” that describes cell line including its
a. name
b. isolation procedure
c. cell culture condition including medi supplements, passage number and
d. seeding density etc.

Thank you for pointing this out. We incorporated the following sentences in the method section of the revised manuscript (Page 18, Line 10-Page 19, Line 7).

Primary cultures of neonatal rat cardiac myocytes

Primary cultures of rat neonatal cardiomyocytes were prepared by the method originally described by Simpson and Savion with minor modifications [5]. Briefly, the hearts from 1- to 3-day-old Wistar rats (Japan SLC Inc., Hamamatsu, Japan) were minced and treated with 0.04% type II collagenase (Sigma) and 0.04% pancreatin (Nacalai Tesque, Kyoto, Japan). The dispersed cells were collected and seeded into gelation-coated dishes at 5 x 10^5 cells/35 mm dish or 1.2 x 10^5 cell/24 mm dish. Cells were incubated in Dulbecco’s modified Eagle’s medium (DMEM/F12) supplemented with 5% horse serum and 10^{-5} mol/L cytosine arabinoside (AraC), which preferentially reduces the proportion of proliferative non-myocytes. Eighteen hours later, the culture medium was exchanged to DMEM/F12 including insulin-transferrin-selenium-A supplement (ITS-A, Invitrogen), 100 U/ml penicillian, 100 μg/mL streptomycin, and 10 mmol/L glutamine. The cells were plated on plastic dishes coated with 1% (w/v) gelatin. Twenty-four hours after seeding, the culture medium was changed to serum-free low-grade DMEM and myocytes were incubated for 24 hours after seeding under humidified 5% CO_2, 95% air incubator at 37°C.

Criticism-5:

Please clarify why 30°C was chosen to conduct ADA mediated deamination study when ADA and V2E, v3E or V5E mixture was incubated for 5 min.

Response-5:

We apologize that the incubation temperature of ADA-mediated deamination was given incorrectly in the original manuscript. We corrected it from 30°C to 27°C in the revised manuscript [6, 7] (Page 16, Line 2).

Regarding the incubation time, we chose 5 min based on preliminary experiments monitored by UV-Vis spectrophotometry at 265 nm [6, 7]. Time-dependent deamination of vidarabine by 0.32U ADA reached a plateau within 10 min at 27ºC (see below). Thus, we set the temperature and time for ADA reaction at 27ºC for 5 min.

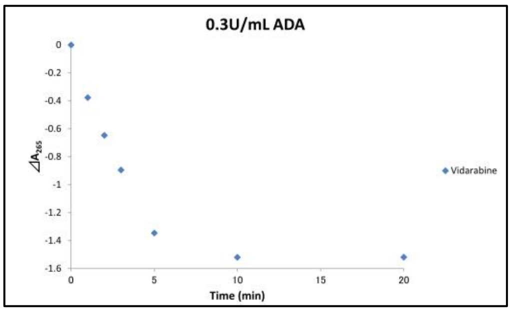

We incorporated following sentences in the method portion of the revised manuscript with new references (Page 16, Lines 1-2).

For ADA treatment, 100 μmol/L vidarabine, V2E, V3E and V5E was preincubated with ADA (0.08, 0.32, 1.25, 5 U/mL) at 27°C for 5min [6, 7].

Criticism-6:

AF induction, why authors waited 10 minutes after IP administration of ISO at 15mg/kg? how this concentration was determined (if adopted, need a ref)

Response-6:

The optimized experimental conditions for AF induction after catecholamine stress using norepinephrine were 10 minutes after IP administration of norepinephrine at 1.5mg/kg [8]. Our preliminary experiments showed that the same experimental conditions were suitable for AF induction after catecholamine stress using ISO instead

of norepinephrine.

We modified the sentences in the method section of the revised manuscript (Page 17, Lines 7-9).

Ten minutes after intraperitoneal administration of 1.5mg/kg ISO (the same conditions as those used for norepinephrine [8, 9]), transesophageal atrial burst pacing was conducted---

Criticism-7:

Measurement of fibrosis area in heart tissue, it is not clear for what was the size/field (as well as its unit) of tissue/images were analyzed to obtain AF area after Masson trichrome staining.

Response-7:

We added the following explanation in the revised manuscript (Page 18, Lines 3-8).

In Masson-trichrome staining, fibers stained in aniline blue were collagen fibers, while those stained in red were muscle fibers. Sections of the cardiac tissues were outlined manually to define regions of interest (ROIs). We measured the percentage fibrosis within 3-5 ROIs for each section, using Image J 1.48v software (National Institute of Health, Bethesda, MD, USA https://imagej.nih.gov/ij/download.html)
[10, 11].

Result section.

In general, according to figure legend each dot in the graph represents individual data. However, it is not clear why some groups have more individual data points than other group. Also, consistency of graph is important for the readers, why some example Fig 4A is bar graph with error bar while showing no individual data points, but Fig 4B shows individual data points. Please clearly state how many animals were used were used in that experiment for each figure (except cell culture study), because it is not clear if individual data points are repeated measurements from single animal or each dot represents different animals.

Response:

We modified **Fig 4A** and **Fig 4B** and incorporated the individual data points in both

figures. We included the number (n) used in the experiments for each figure.
Please also see our response to Criticism-3 from Reviewer #2.

Criticism-8:
What was the half-life of V2E, V3E and V5E?

Response-8:
We thank the reviewer for this interesting question. However, we think this is beyond the scope of the present work and would be difficult to address within a reasonable timeframe for this revision. Thus, we would prefer to leave this characterization for follow-up research to be reported in a subsequent paper.

Criticism-9:
Authors should consider finding better way to present Figure 2-4.

Response-9:
Thank you for the suggestion. We have modified **Figure 2-4** in the revised manuscript to clarify the situation for readers.

Criticism-10:
Fig. 5. For inserted scale bar, it's unit is not described in the figure legend. As mentioned before, it is critical to include number of animal/size of tissues analyzed for that experiment. Provided images does not clearly show dapi positive cells.

Response-10:
Thank you.

Fig 5A. We mentioned the unit of the scale bar in **Fig 5A** in the figure legend in the revised manuscript (Page 59, Line 8).

Fig 6A. We replaced the **Fig 6A** with the revised photo. In addition, we have also added insets with 4x magnified images for clarity in **Fig 6A**.

We incorporated the following sentences in the legend of Fig 6 in the revised manuscript (Page 60, Lines 1-2).

The insets show 4x magnified images. Arrows in insets indicate TUNEL-positive nuclei.

Criticism-11:
Fig 6. TUNEL assay, perhaps it is more informative to plot “number of positive stained cells/dapi positive cells” rather than “Tunel positive (%)” on the Y axis of the graph. Please add scale bar to the figure, if possible.

Response-11:
We have modified the Y axis of the **Fig 6B** as requested and added a scale bar in **Fig 6A**.

Discussion.
Criticism-12:
Eventhough, authors claimed that “AC5KO revealed that genetic disruption of AC5 did not affect cardiac function at baseline [7]”, however their cited study indicated that conscious AC5KO mice showed significantly increased heart rate compared to WT. Therefore, it would be critical to discuss how current result could be affected by this.

Response-12:
Disruption of the AC5 gene has no effect on baseline cardiac function, but leads to an increase in heart rate (HR) despite reduced baseline AC activity. The increased basal HR may be related to a loss of parasympathetic restraint, because AC5 is a Gi-inhibitable AC isoform, although other so-far-unidentified mechanisms may also play a role in regulating baseline cardiac function and HR in AC5KO [12].

We incorporated the above sentences in the discussion of the revised manuscript (Page 33, Line 16-Page 17, Line 3).

Criticism-13:
Authors should discuss why rat neonatal cardiomyocytes were used for apoptotic assay (TUNEL), over mouse derived cardiomyocytes. If not, it should be noted as their experimental limitation(s).

Response-13:
In previous studies, we have demonstrated that viadrabine effectively inhibits

ISO-mediated cardiac myocyte apoptosis in both cultured rat neonatal cardiac myocytes [13] and cultured mouse neonatal cardiac myocytes [14]. However, we agree with the reviewer and have incorporated the following sentences in the discussion portion as a study limitation (Page 38,Lines 11-17).

Limitations
A possible limitation in this study is that the TUNEL study was performed using cultured rat neonatal cardiac myocytes **(Fig 6)**. We have previously demonstrated that viadrabine inhibits ISO-mediated cardiac myocyte apoptosis in both cultured rat neonatal cardiac myocytes [13] and cultured mouse neonatal myocytes [14]. However, further validations using neonatal mouse cardiomyocytes might be desirable in the future.

Criticism-14:
References are not uniformly presented such as .[X] rather than [X].

Response-14:
We have unified the style.

Author contribution.
Criticism-15:
Could you please add name of author who conducted statistical analysis to "Author contribution" section under "statistical analysis" subsection?

Response-15:
PLoS One submission system does not provide the category "statistical analysis" as a contributor role. The PLoS One submission rules state that the authors who performed statistical analysis should be included in "Formal analysis".

Reviewer 3：

Criticism-1:
Authors do not provide enough validation of their synthesized compounds. They provide is a molecular weight for each of the compounds. It is recommended authors should include percent yields, protein NMR, and some sort of mass spectrometry trace, be it from an LC-MS, HPLC, or hi-res mass spectrometer. These are all standard forms

of validation for synthesized compounds, and therefore should be reasonable expectations for the manuscript.

Response-1:

We added percent yield and ^{1}H NMR spectra of V2E **(S5 Fig** of **S1 Data)**, V3E **(S7 Fig** of **S1 data)** and V5E **(S9 Fig** of **S1 data)** in the S1 data and incorporated the following sentences in the method section of the revised manuscript. The NMR data are fully consistent with the expected structure.

1) Page 11, Lines 3-5

Proton nuclear magnetic resonance (^{1}H NMR) data are described in the following order: multiplicity (s: singlet, d: doublet, t: triplet, q: quartet and m: multiplet), coupling constant (J) in Hertz (Hz) and number of protons.

2) Page 11, Lines 11-15

Yield: 90%.^{1}H NMR (300 MHz, DMSO-d_6) δ8.24 (s, 1H), 8.11 (s, 1H), 7.29 (s, 2H), 6.45 (d, J = 6.0 Hz, 1H), 5.84 (d, J = 5.5 Hz, 1H), 5.32 (t, J = 6.0 Hz, 1H), 5.12 (t, J = 5.7 Hz, 1H), 4.46 (q, J = 5.8 Hz, 1H), 3.87-3.61 (m, 3H), 3.05 (d, J = 17.0 Hz, 1H), 2.53 (d, J = 17.2 Hz, 1H), 1.91 (s, 6H). The ^{1}H NMR spectrum of V2E is shown in **S5 Fig** of **S1 Data**.

3) Page 12, Lines 2-6

Yield: 92%.^{1}H NMR (300 MHz, DMSO-d_6) δ8.22 (s, 1H), 8.14 (s, 1H), 7.30 (s, 2H), 6.27 (d, J = 4.4 Hz, 1H), 6.07 (s, 1H), 5.28 (t, J = 3.3 Hz, 1H), 5.22 (s, 1H), 4.31 (s, 1H), 4.00-3.96 (m, 1H), 3.70 (s, 2H), 3.29 (s, 2H), 2.28 (s, 6H). The ^{1}H NMR spectrum of V3E is shown in **S7 Fig** of **S1 Data**.

4) Page 12, Lines 11-15

Yield: 65%. ^{1}H NMR (300 MHz, DMSO-d_6) δ8.14 (s, 1H), 7.26 (s, 2H), 6.30 (d, J = 4.1 Hz, 1H), 5.79 (d, J = 4.4 Hz, 1H), 5.72 (d, J = 4.1 Hz, 1H), 4.41 (dd, J = 7.1, 11.8 Hz, 1H), 4.30 (dd, J = 3.6, 11.8 Hz, 1H), 4.16 (s, 2H), 3.98 (s, 1H), 3.19 (d, J = 2.8 Hz, 2H), 2.23 (s, 6H) The ^{1}H NMR spectrum of V5E is shown in **S9 Fig** of **S1 Data**.

Criticism-2:

Cardiac hypertrophy accompanies many forms of heart disease, including ischemic disease, hypertension, heart failure, and valvular disease. Ventricular hypertrophy is

associated with significantly increased risk of heart failure. In this context cellular calcium homeostasis also plays crucial role. Hence, it is recommended to check the effect of the vidarabine derivatives on calcium homeostasis and cardiac hypertrophy.

Response-2:

We examined the effects of vidarabine and its derivative V2E on cardiac hypertrophy and calcium homeostasis after chronic ISO infusion and incorporated the .following paragraph in the method section of the revised manuscript.

1) V2E ameliorates cardiac hypertrophy after chronic ISO (Page 27, Line 15-Page 28, Line 7)

We examined the effects of vidarabine and V2E on cardiac hypertrophy after chronic ISO treatment **(S11 Fig** of **S1 Data)**. Cardiac hypertrophy as exemplified by ventricular weight per body weight ratio (mg/g) was significantly increased after chronic ISO treatment for 1 week (Control vs. ISO: 4.3 ± 0.3 mg/g vs. 5.2 ± 0.2 mg/g, $P < 0.01$), in accordance with previous findings [15]. However, vidarabine and V2E significantly decreased ISO-induced cardiac hypertrophy (ISO + vidarabine: 4.5 ± 0.1 mg/g, $P < 0.001$ vs. ISO; ISO + V2E: 4.7 ± 0.2 mg/g, $P < 0.01$ vs. ISO) **(S11 Fig** of **S1 Data)**.

These data suggest that vidarbine and V2E might ameliorate cardiac hypertrophy after chronic ISO to comparable extents.

2) V2E ameliorates dysregulation of Ca^{2+} homeostasis after chronic ISO (Page 30, Line 7-Page 31, Line 10)

Since phosphorylation of most Ca^{2+}-handling proteins is altered in many models of experimental HF and AF, which might lead to increased Ca^{2+} release from the sarcoplastic reticulumn and disruption of Ca^{2+} homeostasis, we examined the effects of vidarabine and V2E on protein kinase A-dependent ryanodine receptor 2 (RyR2) phosphorylation (Ser-2808). RyR2 phosphorylation at Ser-2802 was significantly increased after chronic ISO infusion (Control vs. ISO: 100 ± 16% vs. 224 ± 42%, $P < 0.001$). However, this increase was significantly suppressed in vidarabine- or V2E-treated mice (vidarabine: 161 ± 21%, $P < 0.05$ vs. ISO; V2E: 140 ± 20%, $P < 0.05$ vs. ISO) **(S12A Fig** of **S1 Data)**.

Calpain is an intracellular Ca^{2+}-activated protease and an important mediator of the

action of calcium. Calpains are present predominantly in their full-length, unautolysed/unactivated forms at rest. However, calpain appears in autolyzed forms upon disruption of Ca^{2+}-homeostasis during cardiac pathogenesis such as HF, AF, hypertrophy or ischemic reperfusion [11, 16]. We thus examined calpain 1 activity by measuring the level of autolyzed calpain 1 in cardiac tissue and found that calpain 1 autolysis was significantly greater after chronic ISO infusion (Control vs. ISO: 100 ± 4% vs. 145 ± 15%, $P < 0.001$). However, this increase was significantly inhibited in the heart of vidarabine- or V2E-treated mice (vidarabine: 118 ± 5%, $P < 0.05$ vs. ISO; V2E: 120 ± 19%, $P < 0.05$ vs. ISO) **(S12B Fig** of **S1 Data)**.

These data suggest that vidarabine and V2E protect the heart from catecholamine-mediated dysregulation of Ca^{2+} homeostasis to comparable extents.

Criticism-3:

It is well known that the isoform AC5 deletion, results in protecting the heart from cardiomyopathy, chronic catecholamine stress, and chronic pressure overload. On the contrary, the overexpression of isoform AC6 leads to increase d LV function, improved cAMP and Ca^{2+} handling, and may protect the heart from pressure overload-induced systolic and diastolic dysfunction. The author should check the effect of different vidarabine derivatives on the activity of AC6 as well.

Response-3:

We incorporated the following sentences in the results of the revised manuscript (Page 24, Lines 1-13).

V2E, V3E and V5E inhibit AC5 more potently than AC6 in the heart

We have previously demonstrated that there is no compensatory increase in other minor cardiac AC subtypes (AC2, AC3, AC4, AC7, AC9) in AC5KO [12], and AC6 might be the predominant isoform in AC5KO heart. We thus examined the effects of V2E, V3E and V5E on the ISO-stimulated AC activity (50 μM) in the heart of WT and AC5KO to investigate the inhibitory potency towards AC6. The inhibitory effects of V2E **(S10A Fig** of **S1 Data)**, V3E **(S10B Fig** of **S1 Data)** and V5E **(S10C Fig** of **S1 Data)** on ISO (50 μM)-stimulated AC activity in the heart of AC5KO were significantly smaller than in the case of WT mice. (V2E: WT 87.7 ± 5.9% vs AC5KO 94.7 ± 1.7%, $P < 0.05$; V3E: WT 83.9 ± 7.7% vs AC5KO 94.3 ± 2.1%, $P < 0.05$; V5E: WT 89.5 ± 5.9% vs AC5KO 98.5 ± 5.0%, $P < 0.05$).

These data suggests that V2E, V3E and V5E inhibit AC5 more potently than AC6 in the heart, as is the case with vidarabine [17].

<u>Criticism-4:</u>
Numerous profibrotic factors have been identified at the molecular level such as TGFβ, IL11, AngII, which activate gene expression programs for myofibroblast activation. Therapeutic inhibition of profibrotic factor, which is the master-regulator of fibrosis whether mediated by the vidarabine derivative would be significant finding.

<u>Response-4:</u>
We incorporated the following sentences in the discussion of the revised manuscript (Page 37, Lines 3-13).

It is well established that renin secretion by the juxtaglomerular cells (JG cells), in contrast to almost all secretory cells, is inversely related to intracellular calcium concentration [18, 19]. Thus, paradoxically, elevated intracellular calcium is a potent inhibitor of renin release [20] . AC5, which is a calcium-inhibitable AC isoform and is expressed in JG cells, was demonstrated to be the key enzymatic mediator of cAMP stimulation of renin secretion in response to decreased intracellular calcium concentration [20]. More importantly, the selective AC5 inhibitor NKY80, which is also a vidarabine derivative, completely blocks the increase of both cAMP content and renin release in isolated JG cells [21]. Our data, together with the previous findings, suggest that the renin angiotensin system might be, at least to some degree, a therapeutic target of V2E, V3E and V5E for the treatment of CVD.

<u>Criticism-5:</u>
How Author has determined the dose of Vidarabine derivatives

<u>Response-5:</u>
The dose of vidarabine (15mg/kg/day; a dose approved for clinical use in humans) was selected based upon that used in previous studies: this dose did not eliminate the inotropic effects of acute isoproterenol (ISO), did not depress cardiac function at baseline, and retained high selectivity for AC5 [17]. The doses of V2E, V3E and V5E used in this study were set to be equimolar with that of vidarabine.

We incorporated the above sentences in the method of the revised manuscript (Page 14,

Lines 9-13).

References

1. Johnson RA, Shoshani I. Kinetics of "P"-site-mediated inhibition of adenylyl cyclase and the requirements for substrate. J Biol Chem. 1990;265(20):11595-11600. PMID: 2365688.

2. Ishikawa Y, Katsushika S, Chen L, Halnon NJ, Kawabe J, Homcy CJ. Isolation and characterization of a novel cardiac adenylylcyclase cDNA. J Biol Chem. 1992;267(19):13553-13557. PMID: 1618857.

3. Hanoune J, Defer N. Regulation and role of adenylyl cyclase isoforms. Annu Rev Pharmacol Toxicol. 2001;41:145-174. https://doi: 10.1146/annurev.pharmtox.41.1.145. PMID: 11264454.

4. Okumura S, Vatner DE, Kurotani R, Bai Y, Gao S, Yuan Z, et al. Disruption of type 5 adenylyl cyclase enhances desensitization of cyclic adenosine monophosphate signal and increases Akt signal with chronic catecholamine stress. Circulation. 2007;116(16):1776-1783. https://doi: 10.1161/circulationaha.107.698662. PMID: 17893275.

5. Simpson P, McGrath A, Savion S. Myocyte hypertrophy in neonatal rat heart cultures and its regulation by serum and by catecholamines. Circ Res. 1982;51(6):787-801. https://doi: 10.1161/01.res.51.6.787. PMID: 6216022.

6. Saboury AA, Divsalar A, Jafari GA, Moosavi-Movahedi AA, Housaindokht MR, Hakimelahi GH. A product inhibition study on adenosine deaminase by spectroscopy and calorimetry. J Biochem Mol Biol. 2002;35(3):302-305. https://doi: 10.5483/bmbrep.2002.35.3.302. PMID: 12297022.

7. Ajloo D, Saboury AA, Haghi-Asli N, Ataei-Jafarai G, Moosavi-Movahedi AA, Ahmadi M, et al. Kinetic, thermodynamic and statistical studies on the inhibition of adenosine deaminase by aspirin and diclofenac. J Enzyme Inhib Med Chem. 2007;22(4):395-406. https://doi: 10.1080/14756360701229085. PMID: 17847705.

8. Suita K, Fujita T, Cai W, Hidaka Y, Jin H, Prajapati R, et al. Vidarabine, an anti-herpesvirus agent, prevents catecholamine-induced arrhythmias without adverse effect on heart function in mice. Pflugers Arch. 2018;470(6):923-935. https://doi: 10.1007/s00424-018-2121-4. PMID: 29453615.

9. Suita K, Fujita T, Hasegawa N, Cai W, Jin H, Hidaka Y, et al. Norepinephrine-induced adrenergic activation strikingly increased the atrial fibrillation duration through β_1- and α_1-adrenergic receptor-mediated signaling in mice. PLoS One.

2015;10(7):e0133664. https://doi: 10.1371/journal.pone.0133664. PMID: 26203906.
10. Yagisawa Y, Suita K, Ohnuki Y, Ishikawa M, Mototani Y, Ito A, et al. Effects of occlusal disharmony on cardiac fibrosis, myocyte apoptosis and myocyte oxidative DNA damage in mice. PLoS One. 2020;15(7):e0236547. https://doi: 10.1371/journal.pone.0236547. PMID: 32716920.
11. Ito A, Ohnuki Y, Suita K, Ishikawa M, Mototani Y, Shiozawa K, et al. Role of β-adrenergic signaling in masseter muscle. PLoS One. 2019;14(4):e0215539. https://doi: 10.1371/journal.pone.0215539. PMID: 30986276.
12. Okumura S, Kawabe J, Yatani A, Takagi G, Lee MC, Hong C, et al. Type 5 adenylyl cyclase disruption alters not only sympathetic but also parasympathetic and calcium-mediated cardiac regulation. Circ Res. 2003;93(4):364-371. https://doi: 10.1161/01.res.0000086986.35568.63. PMID: 12869393.
13. Iwatsubo K, Minamisawa S, Tsunematsu T, Nakagome M, Toya Y, Tomlinson JE, et al. Direct inhibition of type 5 adenylyl cyclase prevents myocardial apoptosis without functional deterioration. J Biol Chem. 2004;279(39):40938-40945. https://doi: 10.1074/jbc.M314238200. PMID: 15262973.
14. Tsunematsu T, Okumura S, Mototani Y, Ohnuki Y, Jin H, Cai W, et al. Coupling of β1-adrenergic receptor to type 5 adenylyl cyclase and its physiological relevance in cardiac myocytes. Biochem Biophys Res Commun. 2015;458(3):531-535. https://doi: 10.1016/j.bbrc.2015.01.149. PMID: 25677623.
15. Okumura S, Fujita T, Cai W, Jin M, Namekata I, Mototani Y, et al. Epac1-dependent phospholamban phosphorylation mediates the cardiac response to stresses. J Clin Invest. 2014;124(6):2785-2801. https://doi: 10.1172/jci64784. PMID: 24892712.
16. Bukowska A, Lendeckel U, Bode-Böger SM, Goette A. Physiologic and pathophysiologic role of calpain: implications for the occurrence of atrial fibrillation. Cardiovasc Ther. 2012;30(3):e115-127. https://doi: 10.1111/j.1755-5922.2010.00245.x. PMID: 21108772.
17. Iwatsubo K, Bravo C, Uechi M, Baljinnyam E, Nakamura T, Umemura M, et al. Prevention of heart failure in mice by an antiviral agent that inhibits type 5 cardiac adenylyl cyclase. Am J Physiol Heart Circ Physiol. 2012;302(12):H2622-H2628. https://doi: 10.1152/ajpheart.00190.2012. PMID: 22505646.
18. Rong P, Wilkinson-Berka JL, Skinner SL. Renin in thymus, gut, hindlimb, and adrenal of (mRen-2)27 and normal rats: secretion and content studies. Am J Physiol. 1999;277(4):E639-E646. https://doi: 10.1152/ajpendo.1999.277.4.E639. PMID: 10516123.

19. Harding P, Sigmon DH, Alfie ME, Huang PL, Fishman MC, Beierwaltes WH, et al. Cyclooxygenase-2 mediates increased renal renin content induced by low-sodium diet. Hypertension. 1997;29(1 Pt 2):297-302. https://doi: 10.1161/01.hyp.29.1.297. PMID: 9039118.
20. Ortiz-Capisano MC, Ortiz PA, Harding P, Garvin JL, Beierwaltes WH. Decreased intracellular calcium stimulates renin release via calcium-inhibitable adenylyl cyclase. Hypertension. 2007;49(1):162-169. https://doi: 10.1161/01.HYP.0000250708.04205.d4. PMID: 17088449.
21. Ortiz-Capisano MC, Ortiz PA, Harding P, Garvin JL, Beierwaltes WH. Adenylyl cyclase isoform V mediates renin release from juxtaglomerular cells. Hypertension. 2007;49(3):618-624. https://doi: 10.1161/01.HYP.0000255172.84842.d2. PMID: 17190869.
