# Supplementary material for: Water-soluble vidarabine derivatives alleviate catecholamine-induced heart failure and arrhythmia without impairing cardiac function in mice
Source: PLoS One. 2025 Aug 20;20(8):e0330507. doi: 10.1371/journal.pone.0330507 (PMC12367191; doi:10.1371/journal.pone.0330507)
Supplement: Attachment — Submitted filename: PLOS ONE Suita et al. 2nd revise-03.pdf [file pone.0330507.s007.pdf]

Reviewer #2:

Thanks for addressing the raised comment/questions.

A few points from my side:

1) Authors should provide original pictures for all western blots these are shown in the supplementary figure (including molecular weight marker).

Response: I rechecked and confirmed that original pictures for all western blots are provided in the S2 Data. However, original pictures did not contain molecular weight marker. We thus provide original pictures including molecular weight marker for all western blots (Supplementary figure 12 in S1 Data) in S2 Data.

2) Please remove website link from the manuscript (page 18, at the end of subsection "measurement of fibrosis are in heart tissue").

Response: As requested, we have removed the website link.

Reviewer #3:

All the questions asked are almost discussed and experiments are done in detailed manner except some which I could see asked by other reviewer probably beyond scope. Explanation for all the criticism seems reasonable.

Response: Thank you.
